# Cryopreservation of Primary Mouse Neurons: The Benefit of Neurostore Cryoprotective Medium

**DOI:** 10.3389/fncel.2018.00081

**Published:** 2018-03-22

**Authors:** Francesca Pischedda, Caterina Montani, Julia Obergasteiger, Giulia Frapporti, Corrado Corti, Marcelo Rosato Siri, Mattia Volta, Giovanni Piccoli

**Affiliations:** ^1^CIBIO, Dulbecco Telethon Institute, University of Trento, Trento, Italy; ^2^Institute for Biomedicine, EURAC Research, Affiliated Institute of the University of Lübeck, Bolzano, Italy

**Keywords:** neurons, cryopreservation, electrophysiology, 3R, neurites

## Abstract

Primary neuronal culture from rodents is a well-established model to investigate cellular neurobiology *in vitro*. However, for this purpose cell cultures need to be generated expressly, requiring extensive animal handling. Furthermore, often the preparation of fresh culture generates an excess of cells that are ultimately wasted. Therefore the ability to successfully cryopreserve primary neural cells would represent an important resource for neuroscience research and would allow to significantly reduce the sacrifice of animals. We describe here a novel freezing medium that allows long-term cryopreservation of primary mouse neurons prepared from E15.5 embryos. Combining imaging, biochemical and electrophysiological analyses, we found that cryopreserved cultures are viable and mature regarding morphology and functionality. These findings suggest that cryopreserved neurons are a valuable alternative to acutely dissociated neural cultures.

## Introduction

Primary rodent cultures are commonly used in neuroscience studies. Because of the short shelf-life of acutely dissociated culture, cells are normally employed in experiments immediately upon isolation. Another limiting factor is represented by the number of pregnant animals and the time required to reach specific developmental stages. Cryopreservation is a routine technique in both research and clinical settings to allow long-term storage of mammalian cells and tissues such as blood, bone marrow cells, spermatozoa and embryos. A robust protocol to achieve prolonged cryopreservation of neuronal cells would reduce the number of animals sacrificed and the waste of cells. Furthermore, cell storage might allow archiving of cultures, replication of experiments from the same source and facilitate collaboration among different laboratories based at distant locations. The first attempts to achieve nerve tissue cryopreservation were published by Luyet and Gonzales (1953). Since then, several studies have suggested methodologies to cryopreserve neurons and neural stem cells (Ichikawa et al., 2007; Paynter, 2008; Ma et al., 2010). In recent years, different companies have started to commercially provide cryo-preserved neurons. However, this possibility does not help scientists investigating transgenic animals in the context of the study of genetic diseases. Here we describe an innovative freezing medium that allows the long-term storage of primary mouse neurons. We thawed cultures after a 2 or 4 week-long storage at −80°C and we investigated viability, structural and morphological parameters in addition to electrophysiological properties. Our results show that primary mouse cortical cells can be frozen and thawed by standard laboratory procedures without severely affecting their viability or capability to maturate and display characteristic neuronal features.

## Materials and Methods

### Neuronal Cultures, Infection and Drugs

Cortical and hippocampal neuron cultures were prepared from mouse embryos (E15.5; strain C57BL/6J). Tissue specimen was collected in Dissection media (HBSS 1×, 6 mM MgSO_4_, 10 mM HEPES pH 7.4, 10 μg/ml gentamicin) and culture cultivated in Neuronal complete medium (Neurobasal 1×, B27 supplement 1×, 0.5 mM L-glutamine, 10 μg/ml gentamicin). All culture reagents were purchased from Thermo Fischer Scientific. High-density (750–1000 cells/mm^2^) neuronal cultures were plated and grown on 12 mm diameter coverslips put into 24-well plastic tissue culture plates as previously described (Iwaki; Bibby Sterilin; Corti et al., 2008; Pischedda and Piccoli, 2016). In these cultures, glial growth is reduced to less than 0.5% of the nearly pure neuronal population (Brewer et al., 1993). GFP-expressing pLVTH virus was previously described (Bauer et al., 2009). Primary neuronal cultures were transduced at days *in vitro* 4 (DIV4) with viruses at a multiplicity of infection (MOI) 2. Neurons were treated with 10 ng/ml FGFb, 5 ng/ml EGF (all from Peprotech, NJ, USA) for 10 min. All methods involving lentiviruses were performed in accordance with the relevant guidelines and national regulations (D. Lgs. 81/08). All procedures involving animals were approved by Body for the Protection of Animals at the University of Trento and National Agency (autorizzazione 793/2016-PR).

### MTT Reduction Assay

The 3-(4,5-dimethylthiazol-2-yl)-2,5-diphenyltetrazolium bromide (MTT) assay developed by Mosmann (1983) was performed to measure culture viability. Cortical cultures were seeded in a 96-well plate at a concentration of 5 × 10^3^ cell/cm^2^ and incubated at 37°C for 24 h. To perform the assay, MTT was solubilized at a concentration of 5 mg/ml in PBS 1× and added to a final concentration of 0.25 mg/ml in medium. Cells were incubated for 2 h at 37°C. Then, the medium was decanted and formazan precipitates were solubilized in 200 μL of DMSO. The absorbance was measured at 570 nm using a spectrophotometer. Cell viability was expressed setting the control condition as 100%.

### Biochemistry and Antibodies

Neurons were washed in PBS and lysed in RIPA buffer (150 mM NaCl, 50 mM HEPES, 0.5% NP40, 1% Sodium-deoxycholate). After 1 h under mild agitation, the lysate was clarified by centrifugation for 20 min at 16,000 *g*. All procedures were performed at 4°C. Protein samples were measured via standard Bradford assay (Bio-Rad., Hercules, CA, USA). For protein identification and relative quantification via Western blotting, a proper volume of sample containing an equal amount of proteins was diluted with 0.25% 5× Laemmli buffer and loaded onto 10% SDS-PAGE gels; the proteins were transferred onto nitrocellulose membrane (Sigma-Aldrich) at 80 V for 120 min at 4°C.

The primary antibodies were applied overnight in a blocking buffer (20 mM Tris, pH 7.4, 150 mM NaCl, 0.1% Tween 20 and 5% non-fat dry milk); primary antibodies (source in parentheses) included: anti-ERK1/2, anti-P-ERK1/2, anti-AKT, anti-P-AKT, anti- synapsin1–3, anti-GluA1, anti-β-actin, anti-s6 ribosomal protein (Cell Signaling); anti-MAP2 (Abcam). Proteins were detected using the ECL prime detection system (GE Healthcare). Images were acquired with the imaging system ChemiDoc Touch (BioRad) and protein quantification was performed measuring the optical density of the specific bands with ImageLab software (BioRad).

### Immunofluorescence and Quantification

Neuronal cultures were infected with viruses at DIV 1–2. Mitochondrial superoxide content in neurons was characterized at DIV14 by incubation with 2.5 μM Mitosox Red (Molecular Probes, Invitrogen) for 45 min at 37°C. Next, neurons were fixed in 4% paraformaldehyde and 4% sucrose at room temperature. GFP positive neurons were randomly chosen for quantification. The fluorescence images were acquired in blind using a LSM Zeiss 510 confocal microscope with Zeiss 20×, 40× or 63× objective (Karl Zeiss, Jena, Germany) at a resolution of 2048 × 2048 pixels. All the measurements were performed using NeuronStudio^1^. Neurites were automatically traced and quantified by the software in terms of length, number and morphology (Wearne et al., 2005; Rodriguez et al., 2008). Data were then logged and analyzed in Microsoft Excel. For synaptic markers and synapse counts, neurons were similarly fixed at DIV14 and immunostained for MAP2, synapsin1–3 (herein, synapsin) and PSD95. Images were acquired using a Leica SP8-X confocal microscope at 63× at the same resolution. A minimum of 10 neurons per coverslip was imaged. MAP2-positive dendrites were masked using ImageJ (NIH, Bethesda, MA, USA) and synapsin- and PSD95-positive clusters within dendritic masks were quantified using Cell Profiler (Carpenter et al., 2006).

### Electrophysiological Recordings of Cultured Neurons

Whole-cell recordings in voltage- and current-clamp modes were performed in a temperature-controlled recording chamber (35–37°C) mounted on an inverted Eclipse-T*i* microscope (Nikon, Tokyo, Japan) and using a MultiClamp 700B amplifier (Molecular devices, LLC). Voltage- and current-command protocols (indicated below) and data acquisition were performed using pClamp 10.0 software and the Digidata 1550 interface (Molecular Devices, LLC). Data were lowpass-filtered at 3 kHz and sampled at 20 kHz. Patch electrodes, fabricated from thick borosilicate glass capillaries, were made using a Sutter P-1000 puller (Sutter Instruments) to a final resistance of 4–6 MΩ when filled with the intracellular solution containing (in mM): 120 KGluconate, 25 KCl, 10 EGTA, 10 HEPES, 1 CaCl_2_, 4 Mg-ATP, 2 Na-GTP and 4 Na_2_-Phosphocreatine (pH 7.4, adjusted with KOH). Cells were perfused with a Krebs solution containing (in mM): 129 NaCl, 5 KCl, 2 CaCl_2_, 1 MgCl_2_, 30 D-glucose, 25 Hepes; pH 7.3 with NaOH. Voltage-clamp recordings (Vh = −60 mV) of evoked ionic currents were performed by applying a voltage step protocol (from −60 mV to +60 mV, 300 ms of duration) while the spontaneous synaptic activity was acquired in gap-free mode. Miniature excitatory postsynaptic currents (mEPSCs) were recorded in the presence of 200 nM TTX. mEPSC traces (frequency and amplitude) were analyzed by using Clampfit 10.0 Program (Molecular Devices, LLC) with a template matching approach. Only events not superimposed and clearly resolved from the baseline noise were considered. Current-clamp recordings of spontaneous and evoked firing activity were carried out by injecting a hyperpolarizing current to maintain the resting potential of the cells near −60 mV. An online bridge-balance compensation was always performed. Spontaneous action potentials (APs) were recorded in a gap-free mode while the evoked firing activity was evaluated by applying long steps at different current intensities (50 pA increments). Parameters of APs were computed by using Clampfit 10.0 Program. Passive properties of the cells were automatically calculated by Clampex. Series resistance was monitored during the experiments and recordings with changes over 20% of its starting value were discarded.

### Statistical Analysis

All data are expressed as mean ± standard error of the mean (SEM). All data were logged into GraphPad Prism and were analyzed with an unpaired Student’s *T*-test (two classes) or analysis of variance (ANOVA) followed by Tukey’s *post hoc* test (more than two classes). The indication of number of experiment *(n)* and level of significance *(p)* are indicated throughout the text.

## Results

### Neurostore Improves Cell Viability Upon Cryopreservation

**Figure 1:**
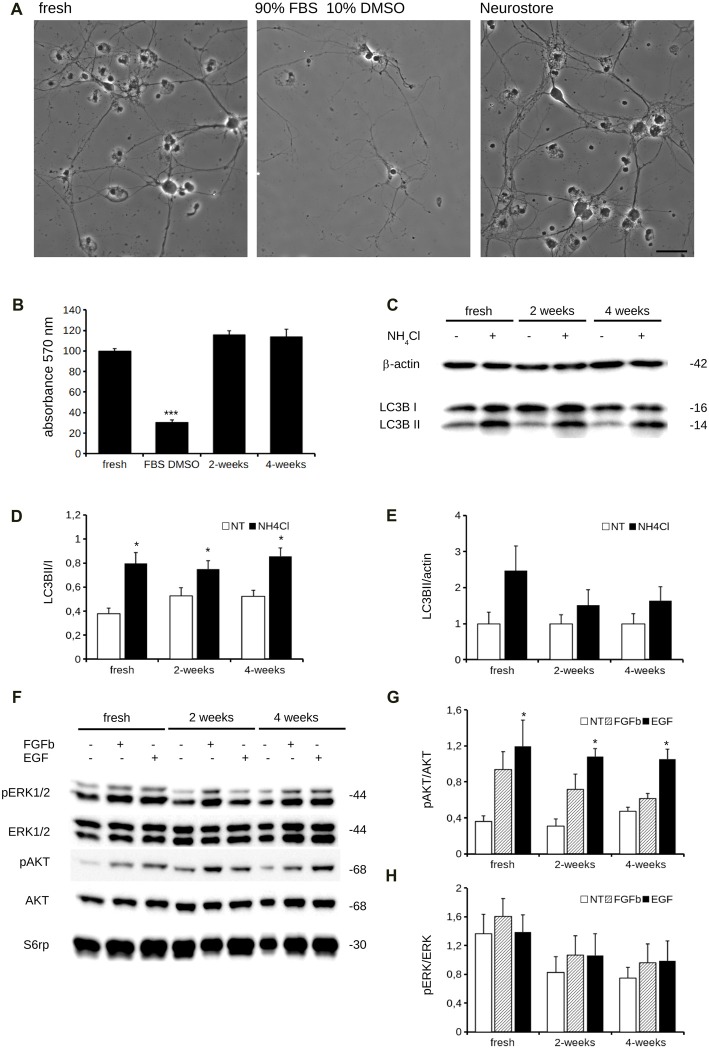
Neurostore improves culture viability after freezing-and-thawing. **(A)** We stored E15.5 mouse cortical neurons for 2-weeks at −80°C in freezing media, containing 90% FBS and 10% DMSO or in Neurostore. After thawing, cells were cultivated for 2 weeks and then imaged under light microscopy. Scale bar = 40 μm. **(B)** We measured viability of acutely dissociated neurons vs. cultures cryopreserved in 10% DMSO and 90% FBS for 2-weeks or in Neurostore for 2 or 4 weeks. We assayed days *in vitro* 14 (DIV14) cultures by 3-(4,5-dimethylthiazol-2-yl)-2,5-diphenyltetrazoliumbromide (MTT). Graph reports absorbance at 570 nm. Data are expressed as mean ± standard error of the mean (SEM), *n* = 6. ***p < 0.001 vs. fresh culture. **(C)** We evaluated autophagic flux in acutely dissociated neurons and cultures cryopreserved for 2 or 4 weeks. Cultures were treated with NH_4_Cl (5 mM, 2 h) at DIV14 and then processed for western-blotting to measure LC3B cleavage. **(D)** The graph reports LC3BII/I optical density ratio. Data are expressed as mean ± SEM, *n* = 6. *p < 0.05 vs. not treated culture. **(E)** The graph reports LC3BII/actin optical density ratio. Data are expressed as mean ± SEM, *n* = 6. **(F)** We evaluated the response to pharmacological stimulation in fresh and cryopreserved cultures. Cultures were treated with 10 ng/ml FGFb or 5 ng/ml EGF for 10 min. at DIV14 and then processed for western-blotting to assess AKT phosphorylation. **(G,H)** The graph reports pAKT/AKT **(G)** and pERK1/2/ERK1/2 **(H)** optical density ratio. Data are expressed as mean ± SEM, *n* = 6. *p < 0.05 vs. not treated culture.

To appreciate the impact of Neurostore on culture viability, we characterized E15.5 mouse cortical cells divided into two parallel experimental groups: they were either directly seeded upon dissection or frozen. To this aim, fixed amounts of neurons 1 × 10^6^) were resuspended in freezing media containing 10% DMSO and 90% FBS or in Neurostore. Cultures were cooled to −80°C in controlled conditions (−1°C/min; see also Table 1). We thawed and seeded cultures after 1 week at −80°C. After cultivation for 14 days, we reported by visual inspection that cultures frozen in Neurostore displayed satisfactory viability and formed a complex network of processes (Figure 1A). Next, we compared the viability at DIV14 of acutely dissociated neurons vs. cultures cryopreserved in 10% DMSO 90% FBS for 2-weeks or in Neurostore for 2 or 4 weeks. By means of MTT assay, we noticed a comparable viability among fresh and cultures stored in Neurostore at −80°C for 2 or 4 weeks (Figure 1B). Accumulating evidence demonstrates that cold stress induces cellular responses including autophagy (Lu and Xu, 2013). Thus, we asked whether the autophagic machinery was up-regulated in cryopreserved cultures. To this aim, we analyzed the autophagic flux in DIV14 acutely dissociated, and 2 or 4-weeks cryopreserved cultures. This process can be monitored by tracking the mobility shift from LC3I to LC3II and LC3II amount by SDS-PAGE. In particular, autophagic flux can be measured by inferring LC3-II turnover in function of lysosomal degradation (Mizushima and Yoshimori, 2007). Thus, acutely dissociated and cryopreserved cultures were either subjected to application of NH_4_Cl (5 mM, 20 min) or left untreated. NH_4_Cl neutralizes lysosomal pH and consequently blocks autolysosomal degradation of LC3II, and thus we then monitored LC3II/LC3I ratio and LC3II relative amount (Figure 1C). Interestingly, we did not observe any major alteration of the autophagic flux in cryopreserved cultures (Figures 1D,E). Functionally competent neuronal cultures respond to pharmacological stimulation by activating specific intracellular pathways. Thus we stimulated DIV14 acutely dissociated and 2- or 4-week cryopreserved cultures with a panel of growth factors and we studied their signaling cascade. In particular, we treated cultures with FGFb (10 ng/ml, 10 min) and EGF (5 ng/ml, 10 min) and we assessed AKT and ERK1/2 phosphorylation (Figure 1F). Noteworthy, cryopreserved cultures react similarly to acutely dissociated ones with regard to AKT or ERK1/2 phosphorylation upon FGFb or EGF stimulation (Figures 1G,H). Altogether these data suggest that Neurostore improves the viability of neuronal cultures upon cryopreservation.

**Table 1:** The protocol illustrates how to freeze and thaw cells stored in Neurostore.

Freezing procedure
1. Warm Neurostore media and cell cooler at 37°C
2. Upon dissection, pellet cells at 600 g 10 min. Average yield is about 8,000,000 cell/cortices, 2,500,000 cells/hippocampi obtained from E15.5 mice embryos.
3. Discard media
4. Gently resuspend cells in warm Neurostore media with a p1000 tip (about 10 times, until complete resuspension of the cells is achieved). Avoid cell clumps formation. Suggested concentration is 5,000,000 cells/ml Neurostore media.
5. Store 1 ml of Neurostore media containing neurons in each cryovial
6. Move cell cooler at −80°C. For long term storage (above 1 month) store cells in liquid nitrogen
Thawing procedure
1. Warm bath, dissection media and neuronal complete culture media at 37°C
2. Move cryovials to 37°C bath
3. Add 9 ml warm dissection media to 1 ml Neurostore containing cells
4. Pellet cells at 600 g 10 min
5. Discard media
6. Gently resuspend cells in 1 ml warm neuronal complete culture media with a p1000 tip (about 10 times, until complete resuspension of the cells is achieved). Avoid cell clumps formation
7. Add warm neuronal complete culture media at the desired concentration
8. Seed cells on proper culture support. We suggest to coat culture support with poli-D-Lysine (mol wt 70,000–150,000 resuspended in ultra-pure water at final concentration 50 μg/ml). Optimal density 50,000–80,000 cells/cm^2^

*Media composition is described in “Materials and Methods” section*.

### Immunocytochemical and Biochemical Characterization of Cryopreserved Cultures

**Figure 2:**
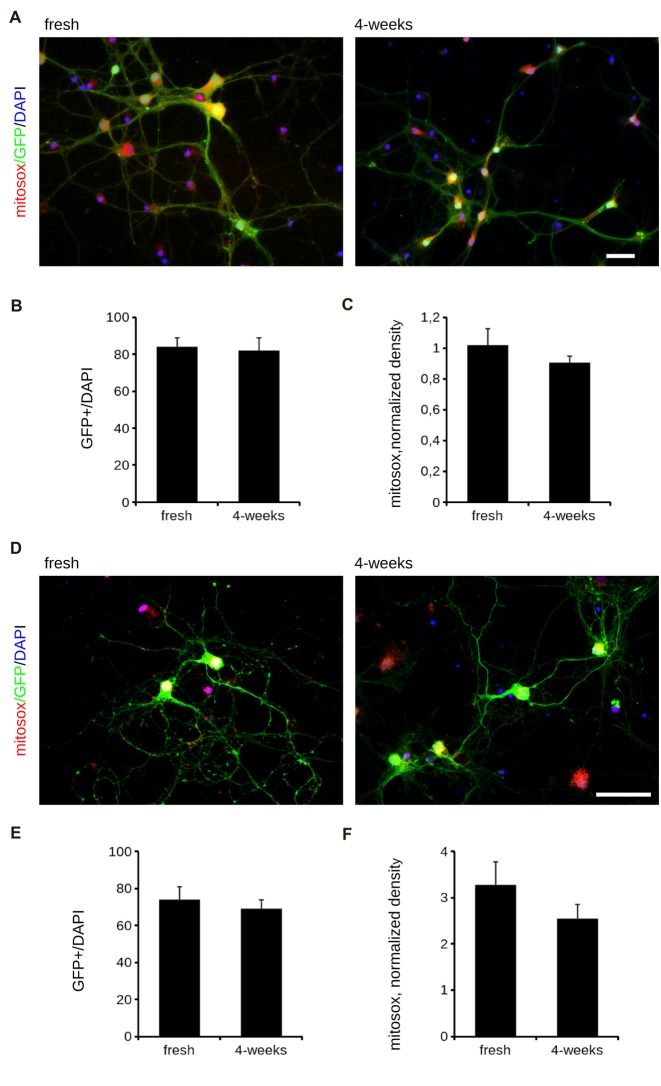
Oxidative stress is comparable between fresh and cryopreserved cultures. We analyzed oxidative stress of acutely dissociated cortical neurons vs. cultures cryopreserved for 2 or 4 weeks.** (A)** Cortical neurons were infected at DIV4 with GFP expressing viruses and then incubated with Mitosox to measure the ROS production at DIV14. Scale bar = 40 μM. **(B)** The graph reports the efficiency of the infection, expressed as ratio between GFP positive cells and total number of nuclei, stained with DAPI. **(C)** The graph reports the intensity of the Mitosox signal, expressed as mitosox integrated density normalized on GFP area (normalized density). Data are expressed as mean ± SEM, *n* = 3 (10 cells/experiment). **(D)** We analyzed oxidative stress of acutely dissociated hippocampal neurons vs. cultures cryopreserved for 2 or 4 weeks. Hippocampal neurons were infected at DIV4 with GFP expressing viruses and then incubated with Mitosox to measure the ROS production at DIV14. Scale bar = 40 μM. **(E)** The graph reports the efficiency of the infection, expressed as ratio between GFP positive cells and total number of nuclei, stained with DAPI. **(F)** The graph reports the intensity of the Mitosox signal, expressed as mitosox integrated density normalized on GFP area (normalized density). Data are expressed as mean ± SEM, *n* = 3 (10 cells/experiment).

**Figure 3:**
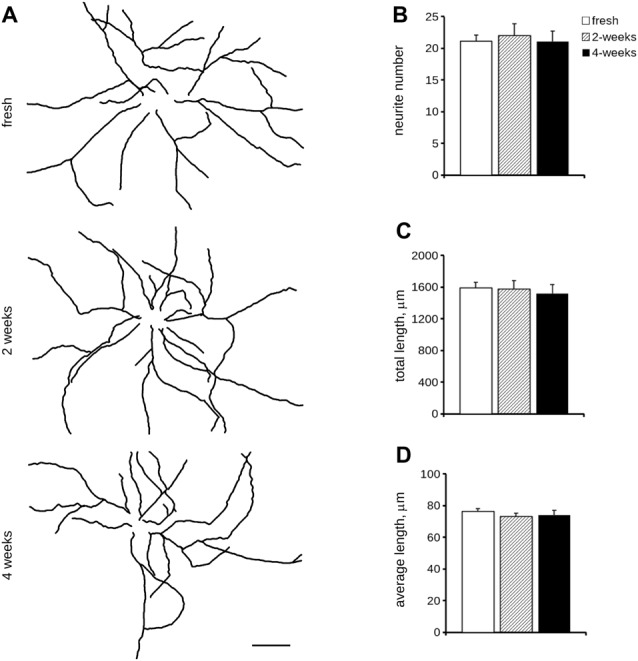
Morphological analysis of cryopreserved cultures. We analyzed morphology of acutely dissociated cortical neurons vs. cultures cryopreserved for 2 or 4 weeks. Neurons were infected at DIV4 and processed for immunofluorescence at DIV14. Panels show camera lucida tracing **(A)**. Graphs show neurite number **(B)**, total length **(C)** and average length **(D)**. Data are reported as mean ± SEM, *n* = 15–22. Scale bar = 50 μm.

**Figure 4:**
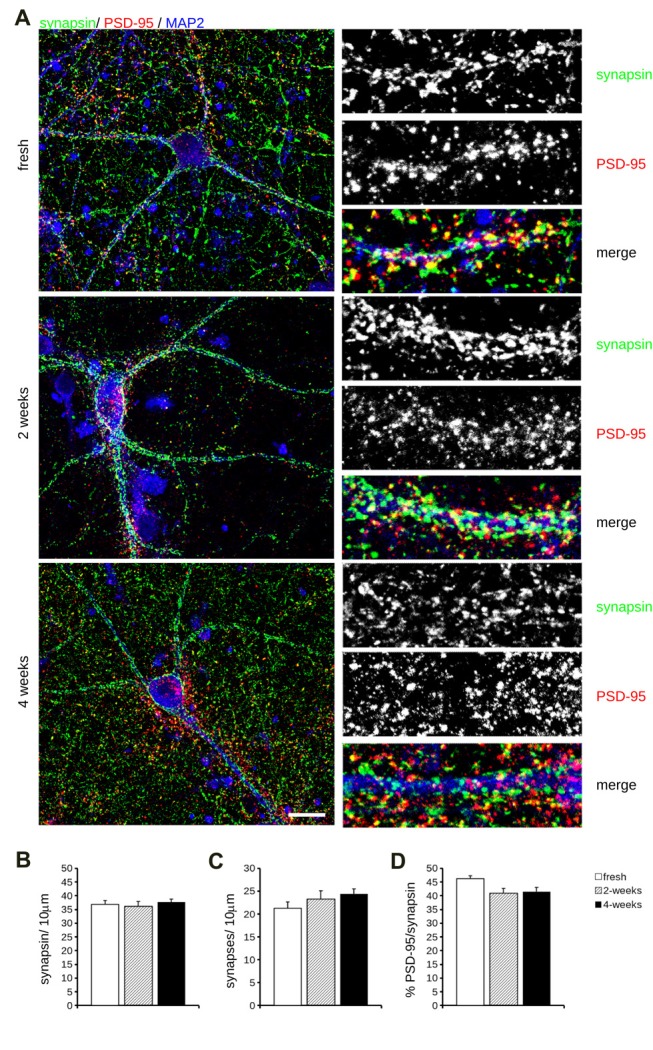
Molecular analysis of cryopreserved cultures.** (A)** We analyzed synaptic composition of acutely dissociated cortical neurons vs. cultures cryopreserved for 2 or 4 weeks. Cultures were fixed at DIV14 and decorated with anti-synapsin (green), anti-PSD-95 (red) and anti MAP-2 (blue) antibodies. Scale bar = 20 μm; boxes show region of interest at higher magnification (12 × 34 μm). **(B–D)** The graphs report the density of synapsin-positive puncta **(B)**, the density of synapses, i.e., puncta positive for both synapsin and PDS-95 **(C)** and fraction of PSD-95 puncta colocalizing with synapsin positive ones **(D)**. Data are expressed as mean ± SEM, *n* = 10–12.

Cryopreservation may induce oxidative stress. Therefore, we measured ROS production in fresh and cryopreserved cortical culture. To this aim, we first infected DIV4 fresh and cryopreserved cultures with GFP expressing viruses. At DIV14, we incubated cultures with MitoSOX Red, a fluorogenic dye selective for mitochondrial superoxide (Figure 2A). Interestingly, both infection efficiency and ROS production resulted similar between fresh and cryopreserved cultures (Figures 2B,C). To further appreciate the impact of Neurostore on culture viability, we characterized E15.5 mouse hippocampal cells divided into two parallel experimental groups: they were either directly seeded upon dissection or frozen in Neurostore for 4-weeks. Next, we measured ROS production in fresh and cryopreserved hippocampal cultures infected at DIV4 with GFP expressing viruses. At DIV14, we incubated cultures with MitoSOX Red (Figure 2D). Interestingly, both infection efficiency and ROS production resulted similar between fresh and cryopreserved cultures (Figures 2E,F). These findings suggest that Neurostore allows long-term storage of hippocampal cultures. During *in vitro* maturation neurons develop a complex tree of processes. Thus, we investigated the morphological development of cortical cultures cryopreserved in Neurostore. To this aim, we first infected acutely dissociated and 2- and 4-week cryopreserved cortical cultures at DIV4 with GFP expressing viruses. At DIV14, we processed cultures for imaging purposes (Figure 3A). The analysis of neurite number, total length and average length did not reveal any significant difference among acutely dissociated and cryopreserved cultures (Figures 3B–D). Mature neuronal processes are decorated by pre and postsynaptic markers. Thus, we immunostained DIV14 acutely dissociated and 2- and 4-week cryopreserved cultures for synapsin and PSD-95, well-established pre- and post-synaptic markers, respectively (Figure 4A). We measured the density of clusters positive for synapsin (Figure 4B), the density of colocalizing clusters (i.e., positive for both synapsin and PSD-95; Figure 4C) which we defined as bona fide synapses (Munsie et al., 2015) and the percentage of PSD-95 positive clusters co-localizing with synapsin positive ones (Figure 4D).

We could not detect any major differences among the experimental groups under analysis.

**Figure 5:**
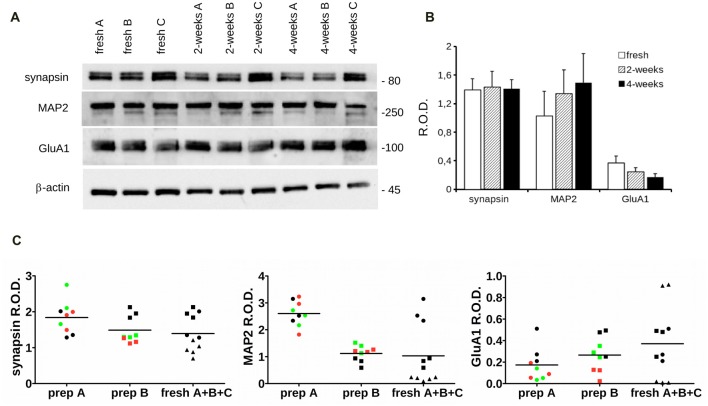
Biochemical analysis of cryopreserved cultures.** (A)** We analyzed the expression level of specific synaptic markers in acutely dissociated cortical neurons vs. cultures cryopreserved for 2 or 4 weeks. Cultures were processed for western-blotting at DIV14 to measure synapsin, MAP2, GluA1 and β-actin levels. **(B)** The graph reports synapsin, MAP2 and Glu1A levels, expressed as relative optical density (ROD) normalized vs. β-actin. Data are expressed as mean ± SEM, *n* = 6. **(C)** The scatter plots show the distribution of single data points obtained from acutely dissociated (black), 2-weeks (green) and 4-weeks (red) cryopreserved cultures coming from two distinct neuronal preparations (prep A and prep B) or combining data from three fresh cultures A (circle), B (square) and C (triangle; fresh A + B + C).

Next, we investigated by western-blotting the expression of key pre and postsynaptic proteins in acutely dissociated and 2- and 4-week cryopreserved cortical cultures at DIV14. In particular, we measured the expression level of synapsin, MAP2 (a cytoskeletal component of the dendrites), AMPA receptor subunit A1 (GluA1) and β-actin (Figure 5A). Our analysis did not report any robust difference in the expression levels of these proteins among acutely dissociated or cryopreserved cultures (Figure 5B). To further test our assessment, we analyzed the distribution of the dataset originating from fresh, 2- and 4-weeks cryopreserved cultures obtained from the same neuron preparation (preparation A or B) or instead combining three independent acute preparations (fresh A + B + C). Noteworthy, the data obtained from independent fresh cultures were more disperse than the ones generated from cultures obtained from the same neuron preparation (Figure 5C). Altogether, these observations suggest that cryopreserved cultures display a physiological maturation at the molecular level.

### Functional Characterization of Cryopreserved Cultures

**Figure 6:**
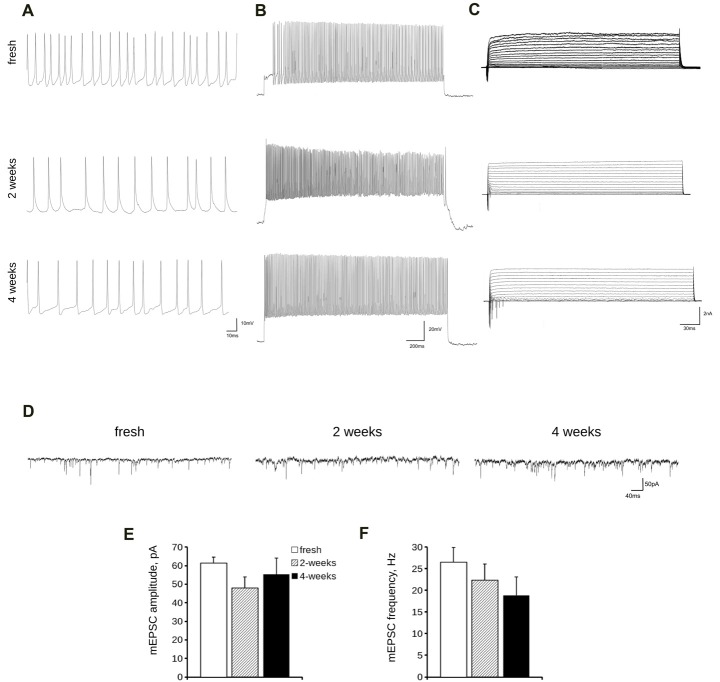
Electrophysiological analysis of cryopreserved cultures. We analyzed passive and active electrical properties of acutely dissociated cortical neurons vs. cultures cryopreserved for 2 or 4 weeks. We measured spontaneous **(A)** and evoked **(B)** firing activity as well as voltage-activated currents **(C)** in DIV14 cultures by whole-cell patch recording. **(D)** We analyzed miniature excitatory post synaptic currents (mEPSC) in acutely dissociated neurons vs. cultures cryopreserved for 2 or 4 weeks. **(E,F)** The graphs report mEPSC frequency **(E)** and amplitude **(F)**. Data are expressed as mean ± SEM, *n* = 8–13.

Lastly, we analyzed the electrical features of acutely dissociated and 2- and 4-week cryopreserved cortical cultures by means of whole-cell patch clamp recording. In particular, we measured passive and active parameters such as resting membrane potential, cell membrane capacitance and spontaneous and evoked firing frequencies (Figures 6A,B) as well as inward and outward voltage-dependent ionic currents (Figure 6C). A quantification of all these parameters demonstrated no significant differences between frozen cultures and fresh neurons (Table 2). The analyses of mEPSC amplitude and frequency (Figures 6D–F) did not reveal any overt differences among fresh and cryopreserved cultures. In conclusion, cryopreserved cultures demonstrated to be fully functional and comparable to freshly prepared neurons.

**Table 2:** The table reports passive and active parameters of fresh and frozen cortical neurons.

Parameters	Fresh	2 weeks	4 weeks
Resting membrane potential (mV)	−56.5 ± 2.4	−59.5 ± 1.4	−57.1 ± 2.5
Cell membrane capacitance (pF)	63.2 ± 6.2	80.3 ± 7.9	79.8 ± 5.8
Input resistance (MΩ)	119 ± 23	106 ± 14	163 ± 31
Maximal firing frequency (Hz)	68.1 ± 9.4	60.3 ± 8.2	64.9 ± 13.1
AP threshold (mV)	−26.5 ± 1.6	−30.3 ± 1.4	−33.7 ± 2.1
AP amplitude (mV)	54.3 ± 1.9	50.0 ± 3.3	56.6 ± 1.5
Half-width (ms)	1.3 ± 0.1	1.4 ± 0.3	1.3 ± 0.2
AHP amplitude (mV)	11.6 ± 0.9	11.9 ± 1.1	11.8 ± 0.8
Overshoot (mV)	27.7 ± 2.4	19.8 ± 3.5	22.5 ± 3.1
Peak Na current (pA/pF)	−79.8 ± 7.5	−62.0 ± 14.2	−95.3 ± 17.1
Peak K current (pA/pF)	116.7 ± 14.0	68.9 ± 17.8	111.1 ± 18.9

*Value are expressed as mean ± SEM, *n* = 7–11; AP, action potential; AHP, after hyper-polarization*.

## Discussion

In this study, we described a simple protocol to achieve long-term storage of primary mouse neurons. Cryopreservation is used as a standard method to store several mammalian cell lines (Grout et al., 1990; Morris, 2007). However, the majority of standard approaches involves tumor cell lines (Odell et al., 2010) or cells immortalized by genetic manipulation with oncogenes (Drayton and Peters, 2002). The vitality of cryopreserved cells strictly depends on cooling rates, fast defrosting, liquid nitrogen long-term-storage and, most important, the usage of cryo-protectants. DMSO is the most common reagent used as cryoprotectant (Yu and Quinn, 1994). Given its low molecular weight, it is capable of permeating cell membranes. It has been suggested that DMSO depresses the freezing point of water, promotes the vitrification of water and eventually prevents the formation of noxious intracellular ice crystals (Mandumpal et al., 2011). Different cryoprotective agents may synergize and improve cell survival after thawing. Neurostore contains a proprietary combination of cryoprotective agents to reach a superior viability of primary cultures upon thawing. Other studies have proposed protocols to achieve a satisfactory cryopreservation of neural cells (Das et al., 1983; Fang and Zhang, 1992; Negishi et al., 2002; Higgins et al., 2011; Quasthoff et al., 2015; Robert et al., 2016). However, previously published studies measured mainly qualitative outcome of viability (Das et al., 1983; Fang and Zhang, 1992; Robert et al., 2016) or cell membrane integrity (Negishi et al., 2002). Few authors characterized cryopreserved cultures in deeper detail. For example, Higgins et al. (2011) provided a qualitative evaluation of neuritic tree formation and only Quasthoff et al. (2015) included a parametric analysis of morphological and functional development of the cultures. In our study, we found no major differences in the viability of the fresh and frozen cells at different time points of their maturation. We further provided measurable outcomes describing the main features of neuronal cultures, including development of neuritic arborization, density of pre and postsynaptic structures and electrophysiological activity. We found no significant differences between cryopreserved or freshly dissected cells regarding electric activity or morphological maturation. Henceforth, despite a freeze and thaw cycle cortical cultures established functional synapses leading to excitatory currents. Furthermore, to the best of our knowledge, our study is the first to describe a method to cryopreserve primary neurons from mouse. Nevertheless, several companies now offer cryo-preserved neurons obtained from wild-type rats or mice. Mouse has developed into the premier mammalian model system in the neuroscience field and allows genetic manipulation to generate models of diseases. Thus, thanks to our protocol, it will be possible to circulate primary cultures prepared from transgenic mouse lines among different laboratories worldwide, dramatically reducing the animal stress and the costs related to the shipment. Indeed, further implementation could be possible: the cooling rate, the number of cells suspended in the cryopreservation medium, the thawing procedure or the overall osmolarity of the freezing medium might improve the final viability of the cultures. In conclusion, we described here a protocol that will allow performing experiments with neuronal cell cultures that are independent from animal breeding and pregnancy or expensive commercially available cells. Our method can lower the time and cost required by experiments and more importantly, will reduce the overall amount of sacrificed animals in accordance to the 3R (replace, reduce, refine) principle.

## Author Contributions

FP, CM, JO and GF performed experiments. CC, MRS and MV analyzed data. FP and GP elaborated the method. MV, CC, MRS and GP wrote the manuscript.

## Conflict of Interest Statement

Neurostore is a proprietary formulation developed by the authors under patent consideration. The authors are willing to distribute Neurostore media to any colleague interested under a proper MTA.

## Footnotes

^1^http://research.mssm.edu/cnic/tools.html
